# Neural oscillations induced by gamma sensory entrainment in cognitive biotype of depression

**DOI:** 10.3389/fpsyt.2025.1586075

**Published:** 2025-05-23

**Authors:** Xiaoning Shi, Hongli Wang, Yingying Zhao, Chenyang Wang, Yongsheng Qi, Michel Gao, Mengping Wei, Gang Wang

**Affiliations:** ^1^ Beijing Key Laboratory of Mental Disorders, National Clinical Research Center for Mental Disorders & National Center for Mental Disorders, Beijing Anding Hospital, Capital Medical University, Beijing, China; ^2^ Advanced Innovation Center for Human Brain Protection & Laboratory for Clinical Medicine, Capital Medical University, Beijing, China; ^3^ WM Therapeutics Co. Ltd, Beijing, China; ^4^ School of Basic Medical Sciences, Capital Medical University, Beijing, China

**Keywords:** major depressive disorder, cognitive biotype, gamma sensory entrainment, electroencephalogram, neural oscillations, individualization

## Abstract

**Aim:**

Abnormal neural oscillations have long been associated with cognition in depression, especially gamma oscillation that participates in neurocircuit function, emotion, and cognition. However, whether gamma sensory entrainment induces alterations in neural oscillations within the cognitive biotype of depression remains unresolved.

**Method:**

The study includes 141 depressed patients in remission. We used Measurement and Treatment Research to Improve Cognition in Schizophrenia (MCCB) to identify the cognitive biotype in depression. Gamma sensory entrainment and spectral power were conducted through Electroencephalogram (EEG). Furthermore, we did correlation analysis to explore the relationship between neural oscillations induced by gamma entrainment with cognitive function in depression.

**Results:**

We enrolled 141 depressed patients in remission, 56 were identified as cognitive biotype. We found that alpha power caused by gamma entrainment with 37 and 48 Hz decreased in cognitive biotype. Specifically, alpha power induced by 37 Hz gamma entrainment decreased alpha power in P3 (*t* =-2.394, FDR = 0.049), P4 (*t* =-2.713, FDR = 0.038), Fp1 (*t* =-2.530, FDR = 0.048), T3 (*t* =-2.689, FDR = 0.038), T5 (*t* =-2.341, FDR = 0.049), F7 (*t* =-2.438, FDR = 0.049), T4 (*t* =-2.764, FDR = 0.038) and Pz (*t* =-2.691, FDR = 0.038) channels in cognitive biotype. While alpha power caused by 48 Hz gamma entrainment showed decreased alpha power only in T4 channel (*t* =-3.135, FDR = 0.04) except other channels in cognitive biotype. Moreover, alpha oscillation induced by 37 Hz gamma entrainment was correlated with working memory in P3, P4, Fp1, T3, T5, F7, T4 and Pz channels; problem solving-ability in T5 channel. Alpha oscillation caused by 48 Hz gamma entrainment showed significant correlation with working memory, problem solving-ability and total scores in T4 channel. Furthermore, we observed that the most optimum stimulus frequency of gamma entrainment eliciting peak responses varied among participants.

**Conclusion:**

Our results confirmed the influence of gamma entrainment by sensory stimuli on neural activity in cognitive biotype of depression, and abnormal alpha power was associated with cognitive function. Furthermore, gamma sensory entrainment with the most optimum stimulus frequency could serve as a potential method to improve cognition in cognitive biotype of depression.

## 1 Introduction

Depression has become the leading cause of burden worldwide, characterized by significant cognitive deficits and neural circuit dysfunction (1, 2). Cognitive biotype of depression was proposed with distinct neural correlates, and functional clinical profile that responds to therapies specifically targeting cognitive dysfunction (3). Therefore, assessment and treatment of cognitive biotype of depression is of great importance which would lead to improved functional outcomes​​​​ (4).

Gamma oscillations are interesting because of their interareal coherence in addition to local regulation (5). Abnormal neural oscillations have long been associated with cognition in depression, especially gamma oscillation (30–90 Hz) that participates in neurocircuit function, emotion, and cognition (6–9). The global electroencephalogram (EEG) coherence in gamma bands of depressed patients was significantly higher than controls, especially in the high gamma band (10). Another EEG study found that subjects with high depressive scores had reduced resting gamma power in the anterior cingulate cortex (11), whereas gamma increased in frontal and temporal regions in a study which subjects with depression performed spatial and arithmetic tasks (12). As for cognition, not only enhanced gamma power is observed in the neocortex and hippocampus during information processing, but also the coherence between the brain areas (13–16).

If gamma oscillation contributes to cognition, then inducing gamma during cognitive tasks should impact on neurocircuit functions and eventually behaviors. Recent studies have employed various types of brain stimulation to induce gamma oscillations (6). Gamma Entrainment Using Sensory Stimulation is an emerging treatment for neuropsychiatric disorders that explored artificially inducement of gamma oscillations using sensory entrainment stimulation (17). Many studies have found that gamma entrainment is involved in cognitive function. Wang et al. reported gamma entrainment rescues cognitive impairment by decreasing postsynaptic transmission after traumatic brain injury (18). Moreover, the frequency of gamma entrainment differs in various research. Gamma entrainment of 40 Hz in temporal-parietal areas improved working memory in humans (19, 20). Similarly, research showed reduced behaviorally-driven gamma before the onset of plaque formation or cognitive decline in a mouse model of Alzheimer’s disease. Optogenetically driving FS-PV-interneurons at gamma (40 Hz) reduced levels of amyloid-*β* (A *β*)_1–40_ and A *β*_1–42_ isoforms (21). While, 80 Hz entrainment coupled to the theta peak in dorsolateral prefrontal cortex by tACS improved working memory (22). Gamma entrainment using audiovisual stimuli alleviates chemobrain pathology and cognitive impairment induced by chemotherapy in mice (23). It is currently unclear which is the most suitable stimulation frequency for gamma stimulation to improve cognition. While 40 Hz is commonly used in neuromodulation studies (e.g., TMS or flicker stimulation), this choice typically derives from animal models of gamma entrainment in sensory processing (24). However, the extension to 33–48 Hz in humans demands explicit rationale based on: slow gamma (30–50 Hz) vs. fast gamma (60–100 Hz) sub-bands are associated with distinct cognitive processes (25). The 33–48 Hz range overlaps with “slow gamma” linked to hippocampal-cortical interactions (26), our target aligns with this mechanism. In depression, specific gamma sub-bands correlate with emotional processing: 40–48 Hz abnormalities in amygdala-prefrontal circuitry, while 30–40 Hz deficits relate to reward processing. Gamma peak frequencies vary inter-individually based on age, genetics, and pathology (27). A fixed 33–48 Hz range risks suboptimal entrainment without individual peak identification (e.g., baseline EEG-guided personalization). The 40 Hz preference in rodents stems from auditory-driven gamma resonance (28), but human cortical gamma exhibits broader natural ranges: 25–45 Hz in resting EEG (29). Broad frequency ranges (15 Hz bandwidth) may inadvertently entrain adjacent beta (20–30 Hz) or high-gamma (>50 Hz) bands due to harmonic interactions. A narrower, biologically constrained range (e.g., 38–48 Hz) could improve target specificity. Therefore, we would like to explore the most appropriate frequency of gamma stimulation in cognitive biotype of depression.

Here, we distinguished cognitive biotype in stable depression by assessing cognition through the MATRICS Consensus Cognitive Battery (MCCB). Moreover, gamma sensory entrainment and spectral power was conducted via EEG, and then we analyzed its correlation with cognitive scores. We aim to investigate the potential biological markers and appropriate frequency of gamma intervention strategies in cognitive biotype of depression.

## 2 Materials and methods

### 2.1 Participants

This study enrolled 141 individuals aged 12–60 in Beijing Anding Hospital with unipolar depression without psychotic symptoms (according to the diagnostic criteria of DSM-5). Visual Analogue Scale score of 5 or above is required to reduce the impact of emotional states. Participants will be excluded if the patients comorbid of compulsive disorder or substance abuse, or who were unable to complete the questionnaire. Subjects who meet any of the following criteria also will be excluded: Those who have undergone electroconvulsive therapy within 3 months prior to enrollment or are currently deemed by investigators to require the treatment; Those receiving concurrent treatments at baseline-including cognitive behavioral therapy, vortioxetine pharmacotherapy (30), transcranial magnetic stimulation, vagus nerve stimulation, or deep brain stimulation and require continuation of these interventions during the study period. The final analysis for the gamma entrainment included 56 cognitive biotype (CI) and 85 no cognitive impairment (NCI) subgroup of depression. Written informed consents were provided by all patients, and the study protocol was approved by the Ethics Committee of Beijing Anding Hospital.

### 2.2 Identify cognitive biotype in depression by MCCB cognitive evaluation

We used MCCB to assess the cognition of depressed patients, which consists of 7 dimensions, including attention, working memory, speed of processing, verbal learning, visual learning, reasoning and problem solving, and social cognition (31). And then computed T-scores adjusted for age, sex and educated years of participants (32). Based on the T-scores of MCCB, we distinguish the cognitive biotype from depression. The criteria for cognitive biotype were two or more cognitive dimensions lower than 40 in MCCB evaluation (33).

### 2.3 Gamma sensory entrainment and scalp encephalographic data acquisition

The steady-state auditory-evoked potentials were measured using a 24-electrode DSI-24 system (Wearable Sensing, San Diego, CA) positioned according to the international 10–20 system, providing uniform coverage across the scalp. There were no background noises present. Throughout the recording session, participants remained relaxed in a quiet, electromagnetically shielded room measuring approximately 10 square meters. The subjects wore EEG recording electrode caps and earphones, were played neuromodulation entrained audio sounds for about 5 minutes with eyes open. The entrained audio sounds consisted of 30 seconds of rest, 320 seconds of gamma oscillations entrained audio playback and 30 seconds of rest. Gamma sensory entrainment includes different stimuli frequency between 33 to 48 Hz, 20 second/per frequency. The whole process lasted for approximately 7 minutes. The EEG recordings had a sample rate of 300 Hz, and the signal was average referenced.

### 2.4 Power spectrum analysis of neural oscillations

Power spectral densities (PSD) of neural oscillation were analyzed by EEGLAB (https://sccn.ucsd.edu/eeglab/index.php) (34), a toolbox for MATLAB (The MathWorks, Inc., Natick, MA). The raw EEG data were acquired at a sampling rate of 300 Hz. To extract specific spectral power within each frequency band, the data were first filtered with a high-pass filter at 1 Hz and a low-pass filter at 90 Hz. Additionally, a notch filter was applied at 50 Hz to remove power line interference and then segmented into epochs of 2 seconds. Bad epochs were identified and removed, and bad channels were interpolated using spherical spline interpolation. Independent component analysis (ICA) was performed to decompose the neural oscillation data, which were carefully visually inspected to remove artifacts such as eye blinks, eye movements, body movements, and electrocardiogram interference. After ICA and baseline correction, the recordings were referenced to the average of all EEG channels. Epochs exceeding the amplitude threshold of -100 to 100 µV were rejected. The average spectral power (µV²) of neural oscillations in the standard frequency ranges was calculated using the Welch averaged periodogram method (1). Spectrograms were generated using a Fast Fourier Transform with a Rectangular Window, averaging across the entire epoch for each frequency band. The gamma sensory entrainment-induced power markers were derived into the following frequency bands: delta (δ = 1–4 Hz), theta (θ = 4–8 Hz), alpha (α = 8–13 Hz), beta (β = 13–30 Hz), and gamma (γ > 30 Hz) (35, 36).

### 2.5 Alpha neural oscillations asymmetric analysis

Alpha neural oscillation asymmetry was calculated in 37 Hz and 48 Hz gamma sensory entrainment, respectively. The recordings were re-referenced to Cz, and asymmetry scores were calculated using the normalized power difference between homologous right- and left-side locations, expressed as (R−L)/(R+L) (37). Additionally, alpha oscillation asymmetry was examined in six specific regions: prefrontal [Fp: (Fp2-Fp1)/(Fp2+Fp1)], frontal [F: (F4-F3)/(F4+F3)], central [C: (C4-C3)/(C4+C3)], Temporal [T: (T4-T3)/(T4+T3)], parietal [P: (P4-P3)/(P4+P3)] and occipital [O: (O2-O1)/(O2+O1)] cortex. This index reflects the relative activation of right-side compared to left-side locations in individuals with depression.

### 2.6 Statistical analysis

Data are expressed as mean ± standard deviation (SD). Statistical analyses were conducted using SPSS software (version 28.0), with a level of significance that was set at *P < 0.05, **P < 0.01 and ***P < 0.001. The Independent-sample T-test was used to analyze differences of MCCB scores and power spectral density (including alpha neural oscillation asymmetry) induced by gamma sensory entrainment between the CI and NCI groups. In detail, the independent-sample T-test (FDR adjusted) was used to test differences in alpha power spectral density between two subgroups. Pearson correlation analysis was utilized to explore the relationship of the alpha power spectral density induced by gamma sensory entrainment and cognition in cognitive biotype of depression.

## 3 Results

### 3.1 The effects of gamma sensory entrainment on neural oscillations in cognitive biotype of depression

**Figure 1:**
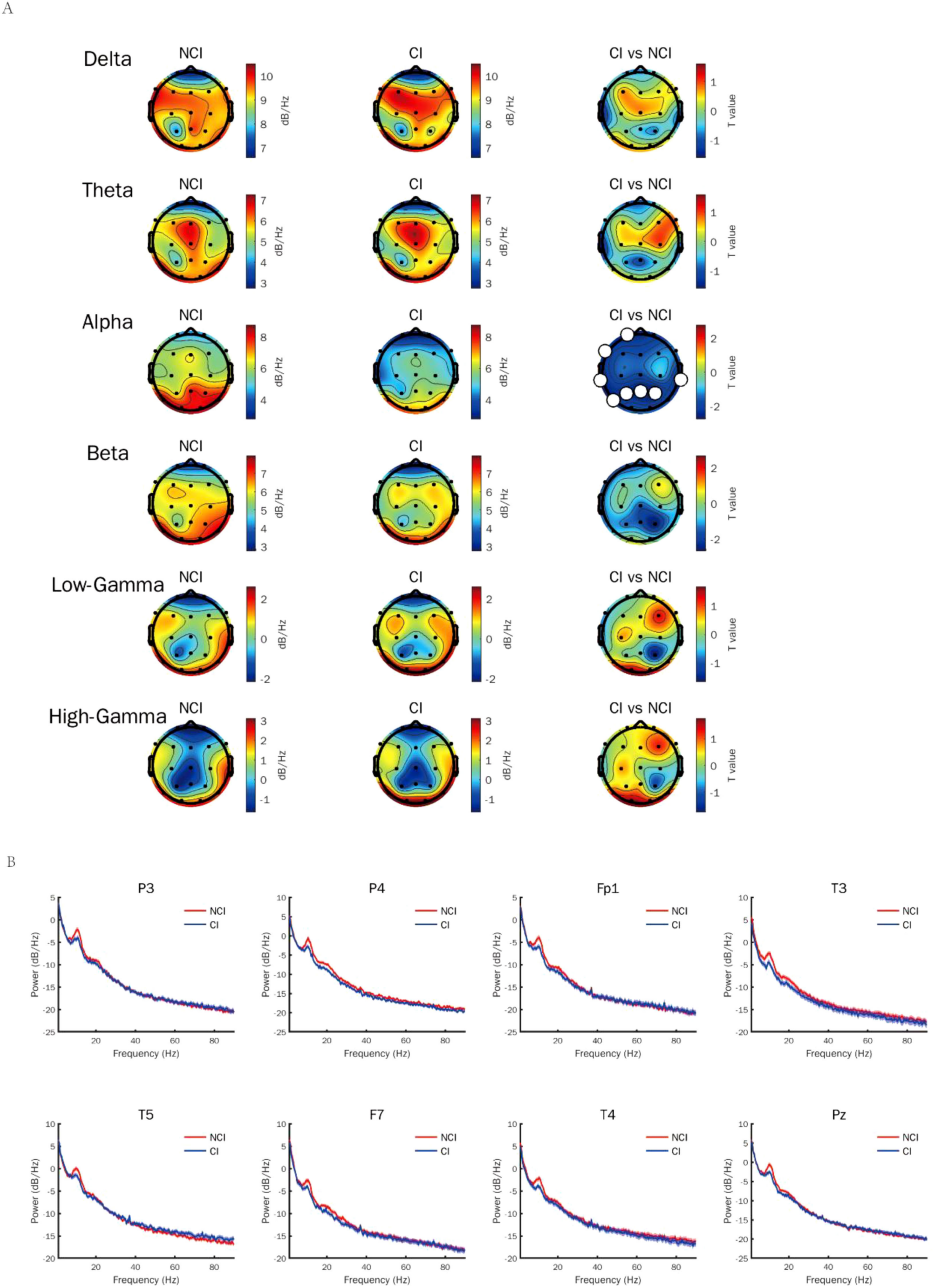
Alpha power induced by gamma entrainment with 37 Hz decreased in cognitive biotype of depression. **(A)** Alpha power except other neural oscillations induced by gamma entrainment decreased in cognitive biotype of depression. **(B)** Alpha power induced by gamma entrainment decreased in P3, P4, Fp1, T3, T5, F7, T4 and Pz channels in cognitive biotype. CI, cognitive impairment group; NCI, non-cognitive impairment.

**Figure 2:**
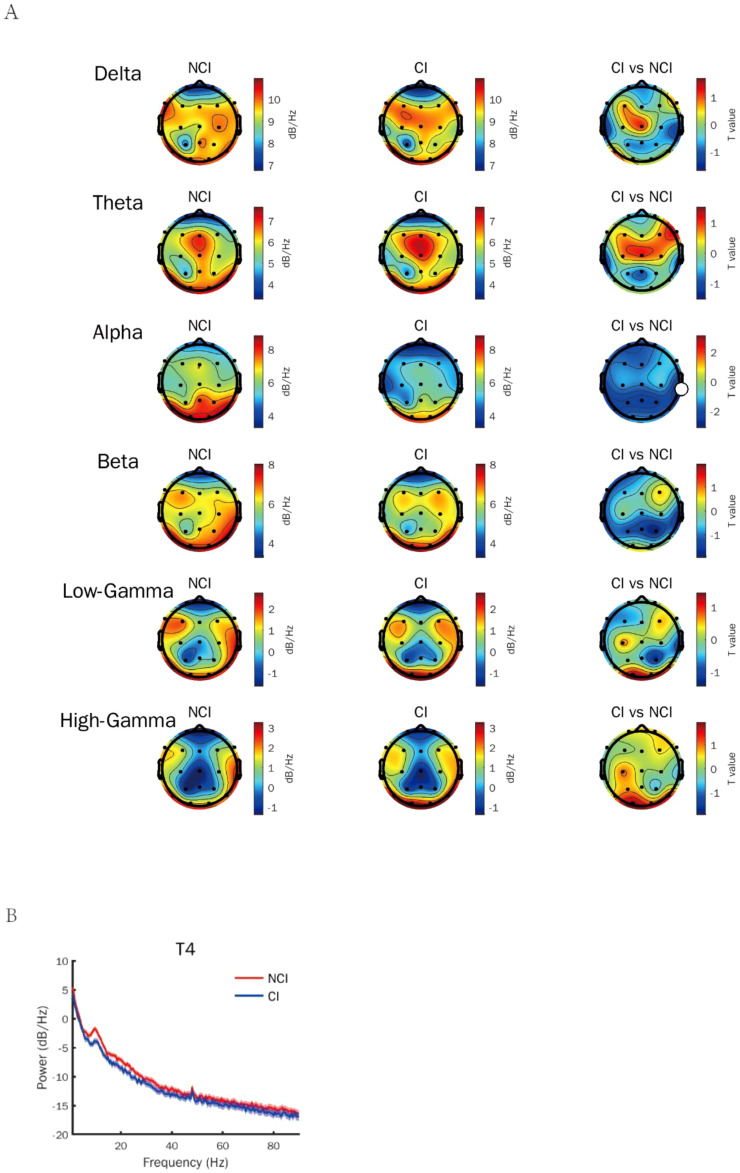
Alpha power induced by gamma entrainment with 48 Hz decreased in cognitive biotype of depression. **(A)** Alpha power except other neural oscillations induced by gamma entrainment decreased in cognitive biotype of depression. **(B)** Alpha power induced by gamma entrainment decreased in T4 channel in cognitive biotype. CI, cognitive impairment group; NCI, non-cognitive impairment.

To identify the most appropriate stimuli frequency of gamma entrainment in cognitive biotype of depression, we calculated the power spectral density of neural oscillations induced by different gamma sensory entrainment between 33 to 48 Hz. Compared to NCI, gamma power existed no difference induced by different gamma sensory entrainment in cognitive biotype (**Table 1**). Interestingly, we found that alpha power caused by gamma entrainment with 37 and 48 Hz decreased in cognitive biotype. Specifically, alpha power caused by 37 Hz gamma entrainment decreased in P3 channel (*t* =-2.394, FDR = 0.049), P4 channel (*t* =-2.713, FDR = 0.038), Fp1 channel (*t* =-2.530, FDR = 0.048), T3 channel (*t* =-2.689, FDR = 0.038), T5 channel (*t* =-2.341, FDR = 0.049), F7 channel (*t* =-2.438, FDR = 0.049), T4 channel (*t* =-2.764, FDR = 0.038) and Pz channel (*t* =-2.691, FDR = 0.038) in cognitive biotype (**Figure 1**). While 48 Hz gamma entrainment showed decreased alpha power in T4 channel (*t* =-3.135, FDR = 0.04) except other channels in cognitive biotype (**Figure 2**). However, other neural oscillation frequency brought by gamma entrainment exhibited no significant differences in two subgroups (FDR > 0.05).

**Table 1:** Effects of gamma sensory entrainment on neural oscillations in depression.

Gamma Entrainment	Channel	T-Value	P-Value	FDR
37 Hz	P3	-2.394	0.018	0.049
	P4	-2.713	0.008	0.038
	Fp1	-2.530	0.013	0.048
	T3	-2.689	0.008	0.038
	T5	-2.341	0.021	0.049
	F7	-2.438	0.016	0.049
	T4	-2.764	0.006	0.038
	Pz	-2.691	0.008	0.038
48 Hz	T4	-3.135	0.002	0.040

### 3.2 Alpha power symmetrically induced by gamma entrainment in cognitive biotype of depression

**Figure 3:**
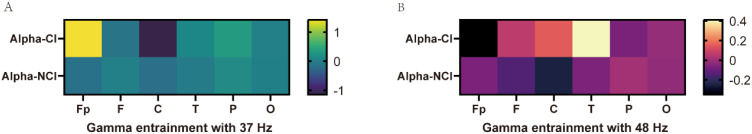
The asymmetry of alpha power values induced by gamma entrainment in CI and NCI biotype in MDD. **(A)** There was no statistical difference in alpha power induced by 37 Hz gamma entrainment between CI and NCI biotypes. **(B)** There was no statistical difference in alpha power induced by 48 Hz gamma entrainment between CI and NCI biotypes. CI: cognitive impairment group, NCI: non-cognitive impairment.

Furthermore, we analyzed the alpha asymmetry induced by gamma entrainment in cognitive biotype of depression. While, there was no statistical difference in alpha power induced by 37 Hz gamma entrainment in Fp (*t* = 1.016, P = 0.314), F (*t* =-0.715, P = 0.476), C (*t* =-1.135, P = 0.261), T (*t* =0.651, P = 0.516), P (*t* =1.315, P = 0.194), O (*t* =-0.567, P = 0.572) and 48 Hz gamma entrainment in Fp (*t* =-1.331, P = 0.186), F (*t* =1.324, P = 0.188), C (*t* =1.348, P = 0.18), T (*t* =1.101, P = 0.275), P (*t* =-0.486, P = 0.629), O (*t* =0.08, P = 0.936) (**Figure 3**, **Table 2**).

**Table 2:** Alpha asymmetry induced by gamma entrainment in depression.

Gamma Entrainment	Alpha Asymmetry	T-Value	P-Value
37 Hz	Fp	1.016	0.314
	F	-0.715	0.476
	C	-1.135	0.261
	T	0.651	0.516
	P	1.315	0.194
	O	-0.567	0.572
48 Hz	Fp	-1.331	0.186
	F	1.324	0.188
	C	1.348	0.180
	T	1.101	0.275
	P	-0.486	0.629
	O	0.080	0.936

### 3.3 The correlation of alpha power induced by gamma entrainment with cognitive function in depression

**Figure 4:**
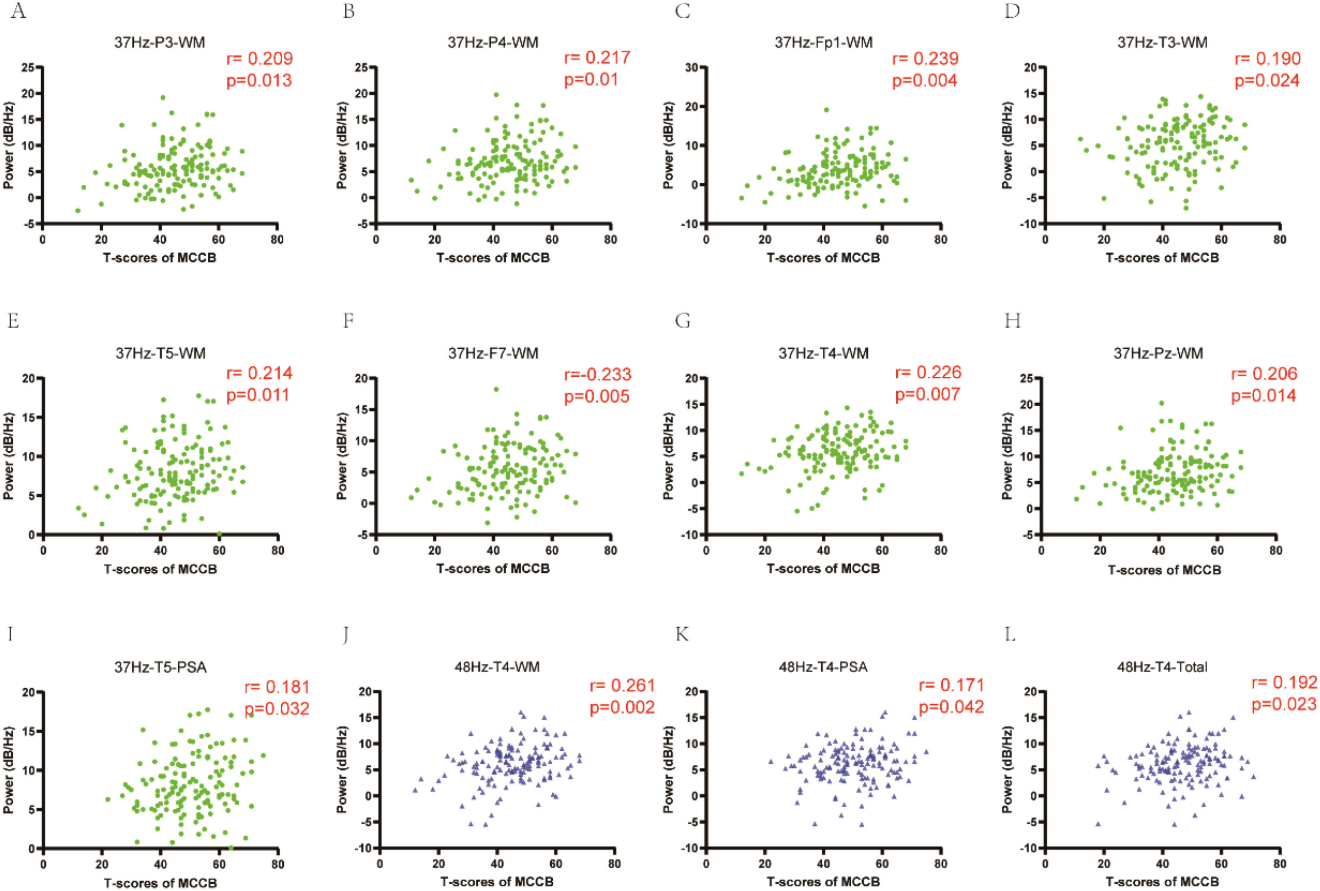
The correlation of alpha power induced by gamma entrainment with cognitive function in depression. **(A-I)** In 37 Hz condition, alpha oscillation induced by gamma entrainment was correlated with working memory in P3, P4, Fp1, T3, T5, F7, T4 and Pz channels; problem solving-ability in T5 channel. **(J-L)**: In 48 Hz condition, alpha oscillation induced by gamma entrainment showed significant correlation with working memory, problem solving-ability and total score in T4 channel. CI, cognitive impairment group; NCI, non-cognitive impairment; WM, working memory; PSA, problem solving-ability.

To further explore the relationship between alpha power induced by gamma entrainment and cognitive function in depression, we did correlation analysis. Significant correlations between cognitive function and alpha power were observed. In 37 Hz condition, alpha oscillation induced by gamma entrainment was correlated with working memory in P3 channel (*r* = 0.209, P = 0.013), P4 channel (*r* = 0.217, P = 0.01), Fp1 channel (*r* = 0.239, P = 0.004), T3 channel (*r* = 0.190, P = 0.024), T5 channel (*r* = 0.214, P = 0.011), F7 channel (*r* = 0.233, P = 0.005), T4 channel (*r* = 0.226, P = 0.007), Pz channel (*r* = 0.206, P = 0.014); problem solving-ability in T5 channel (*r* = 0.181, P = 0.032). While alpha oscillation caused by 48 Hz gamma entrainment showed significant correlation with working memory (*r* = 0.261, P = 0.002), problem solving-ability (*r* = 0.171, P = 0.042) and total score (*r* = 0.192, P = 0.023) in T4 channel except other channels (**Figure 4**).

### 3.4 Individualized peak distribution of neural oscillations induced by gamma sensory entrainment in depression

**Figure 5:**
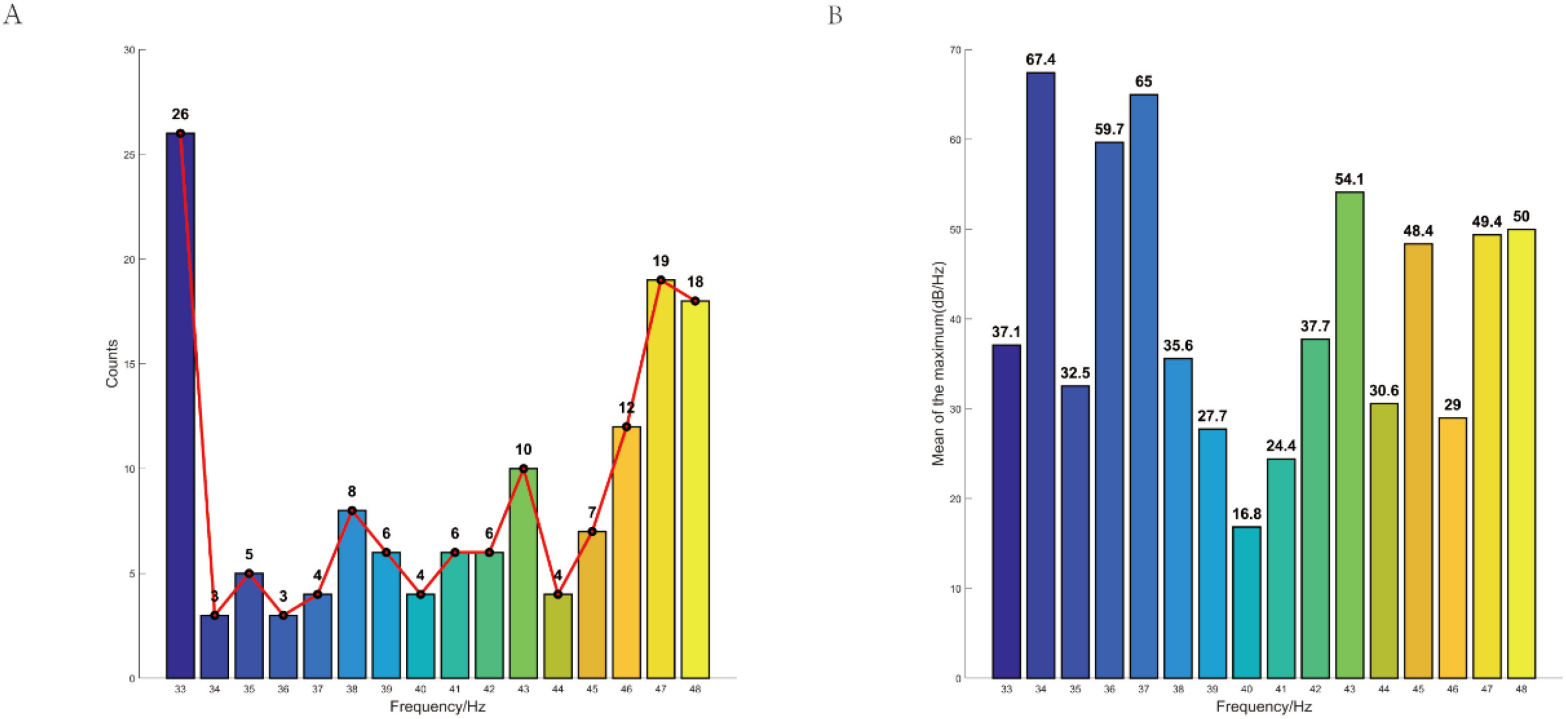
Individualized peak distribution of neural oscillations induced by gamma sensory entrainment in depression. **(A)** The majority of depressive individuals exhibited peak responses at 33 Hz gamma entrainment. **(B)** No significant differences were observed in the average of the maximum neural oscillation elicited by appropriate gamma entrainment among participants. CI, cognitive impairment group; NCI, non-cognitive impairment.

Moreover, we compared the stimulus frequency of gamma entrainment that elicited peak neural responses in each individual. Interestingly, we observed that the stimulus frequency of gamma entrainment eliciting peak responses varied among participants. The majority of depressive individuals exhibited peak responses at 33 Hz gamma entrainment. No significant differences were observed in the average of the maximum neural oscillation elicited by appropriate gamma entrainment among participants (**Figure 5**).

## 4 Discussion

This study suggests that alpha power caused by gamma entrainment with 37 and 48 Hz decreased in cognitive biotype. Specifically, Alpha power induced by 37 Hz gamma entrainment decreased in parietal, left Frontal, temporal, left frontal-temporal regions in cognitive biotype of depression. Moreover, all these alpha oscillations were correlated with working memory, but only problem solving-ability in left posterior temporal lobe. While alpha power caused by 48 Hz gamma entrainment showed decreased alpha power only in right temporal region in cognitive biotype. And it has significant correlation with working memory, problem solving-ability and total score. Furthermore, we observed that the stimulus frequency of gamma entrainment eliciting peak responses varied among participants.

Oscillatory activity in neuronal cells has been associated with a variety of perceptual, motor, and cognitive functions (7, 38–40). Modulation of gamma oscillations as a possible therapeutic tool for neuropsychiatric diseases (41–43). The impact of gamma entrainment on gamma power in depression with cognitive impairment or not was generally similar. Gamma entrainment primarily induces neural activity in the gamma frequency band. However, due to the complexity of brain oscillations and cross-frequency coupling, it may also indirectly affect other frequency bands, such as theta, alpha, beta, etc (44, 45). The study reports a decrease in alpha power following gamma entrainment at 37 Hz across multiple areas in the cognitive biotype of depression. These results suggest that 37 Hz gamma entrainment has a widespread neuromodulatory effect, potentially influencing regions involved in cognitive processing and memory. In contrast, 48 Hz gamma entrainment showed a more localized reduction in alpha power, primarily in right temporal region. This frequency-specific and region-specific effect aligns with previous studies demonstrating that gamma entrainment can selectively modulate neural activity depending on the frequency and brain region targeted (46, 47). More interestingly, research also reported there is a suppression of alpha rhythms with 40 Hz square wave sounds (48). Gamma entrainment is thought to synchronize neural activity, enhancing communication between brain regions involved in cognitive processing (49).

The reduction in alpha power observed in this study may reflect a shift in neural resources from idling (associated with alpha oscillations) to active processing (associated with gamma oscillations). Alpha oscillations are theorized to reflect cortical inhibition mechanisms (50), top-down attention regulation (51), and cross-frequency coupling dynamics (52). Reduced alpha power may indicate impaired inhibitory control over task-irrelevant cortical regions. In depression, this could manifest as hyperactivity in the default mode network (DMN) during rest (53), potentially linked to rumination. A reference to Berger’s original thalamocortical gating theory and modern DMN-alpha interactions would help ground this interpretation. Moreover, alpha oscillations modulate sensory gain in visual/auditory cortices during attentional tasks (54). The observed alpha reduction might reflect failure to suppress distractors (e.g., negative emotional stimuli), consistent with depression’s attentional bias. Linking alpha power to behavioral measures like emotional Stroop performance could bridge neural and cognitive levels.

Depression-related alpha suppression may disrupt cross-frequency coupling with gamma oscillations, which coordinates distributed neural assemblies for cognitive-emotional integration (5). Hipp et al. demonstrated that alpha phase modulates gamma amplitude during working memory-a mechanism potentially compromised in depression (55). Reduced alpha could reflect abnormal thalamocortical resonance, as proposed in models linking mood disorders to disrupted oscillatory hierarchies. The differential effects of 37 Hz and 48 Hz gamma entrainment suggest that specific frequencies may engage distinct neural mechanisms. This could be leveraged in therapeutic interventions to target specific cognitive biotype in depression.

Alpha oscillations induced by 37 Hz gamma entrainment were closely correlated with working memory in multiple areas and with problem-solving ability in the left posterior temporal lobe. This is consistent with research linking alpha oscillations to cognitive control and memory processes (50, 54). For 48 Hz gamma entrainment in right temporal region, significant correlations were observed with working memory, problem-solving ability, and total cognitive score. The results highlight the importance of the right temporal region in mediating the cognitive effects of 48 Hz entrainment, which may reflect its role in integrating sensory and cognitive information (56). The correlation between alpha oscillations and cognitive performance underscores the potential of gamma entrainment as a tool for enhancing cognitive function in depression. It suggests that gamma entrainment may facilitate the reorganization of neural networks, improving efficiency in cognitive tasks. This is supported by studies showing that gamma oscillations play a critical role in information processing and synaptic plasticity (29). Auditory entrainment coordinates cortical-BNST-NAc triple time locking to alleviate the depressive disorder (57). Adaikkan et al. found that Tau P301S and CK-p25 mice subjected to gamma entrainment from the early stages of neurodegeneration showed a preservation of neuronal and synaptic density across multiple brain areas and modified cognitive performance (58). However, the frequency- and region-specific effects suggest that therapeutic efficacy may depend on the targeted cognitive domain and underlying neural circuitry.

Also, the study observed variability in the stimulus frequency of gamma entrainment eliciting peak responses among participants. This variability could be attributed to individual differences in neural oscillatory patterns, brain connectivity, or the severity of depressive symptoms (59). Individual differences in peak response frequencies highlight the need for personalized approaches in gamma entrainment-based therapies. This aligns with the growing emphasis on precision medicine in psychiatry (60). Identifying individual-specific optimal frequencies for gamma entrainment could enhance the effectiveness of interventions and improve outcomes in depression treatment.

The study focused on the cognitive biotype of depression, which may limit the generalizability of the findings to other biotypes or clinical populations. Future studies should explore the effects of gamma entrainment across different subtypes of depression. The mechanisms underlying the frequency-specific effects of gamma entrainment on alpha power and cognitive functions remain unclear. Future research could investigate the role of neurotransmitter systems (e.g., GABA, glutamate) and functional connectivity in mediating these effects (61). Longitudinal studies are needed to determine whether gamma entrainment can produce sustained improvements in cognitive function in depression.

This study provides valuable insights into the neuromodulatory effects of gamma entrainment on alpha oscillations and cognitive function in the cognitive biotype of depression. The findings suggest that gamma entrainment, particularly at 37 Hz and 48 Hz, may serve as a promising therapeutic tool for addressing cognitive deficits in depression. However, the variability in individual responses underscores the importance of personalized approaches in optimizing the efficacy of such interventions. Further research is needed to elucidate the underlying mechanisms and to explore the long-term benefits of gamma entrainment in clinical populations.

## Data Availability

The raw data supporting the conclusions of this article will be made available by the authors, without undue reservation.
